# Natural reassignment of CUU and CUA sense codons to alanine in *Ashbya* mitochondria

**DOI:** 10.1093/nar/gkt842

**Published:** 2013-09-17

**Authors:** Jiqiang Ling, Rachid Daoud, Marc J. Lajoie, George M. Church, Dieter Söll, B. Franz Lang

**Affiliations:** ^1^Department of Molecular Biophysics and Biochemistry, Yale University, New Haven, CT 06520-8114, USA, ^2^Département de Biochimie, Centre Robert-Cedergren, Université de Montréal, 2900 Boulevard Edouard Montpetit, Montréal, Québec, H3C 3J7, Canada, ^3^Program in Chemical Biology, Harvard University, Cambridge, MA 02138, USA, ^4^Department of Genetics, Harvard Medical School, Boston, MA 02115, USA and ^5^Department of Chemistry, Yale University, New Haven, CT 06520-8114, USA

## Abstract

The discovery of diverse codon reassignment events has demonstrated that the canonical genetic code is not universal. Studying coding reassignment at the molecular level is critical for understanding genetic code evolution, and provides clues to genetic code manipulation in synthetic biology. Here we report a novel reassignment event in the mitochondria of *Ashbya (Eremothecium) gossypii*, a filamentous-growing plant pathogen related to yeast (Saccharomycetaceae). Bioinformatics studies of conserved positions in mitochondrial DNA-encoded proteins suggest that CUU and CUA codons correspond to alanine in *A. gossypii*, instead of leucine in the standard code or threonine in yeast mitochondria. Reassignment of CUA to Ala was confirmed at the protein level by mass spectrometry. We further demonstrate that a predicted 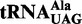 is transcribed and accurately processed *in vivo*, and is responsible for Ala reassignment. Enzymatic studies reveal that 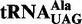 is efficiently recognized by *A. gossypii* mitochondrial alanyl-tRNA synthetase (*Ag*AlaRS). AlaRS typically recognizes the G3:U70 base pair of tRNA^Ala^; a G3A change in *Ashbya* 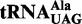 abolishes its recognition by *Ag*AlaRS. Conversely, an A3G mutation in *Saccharomyces cerevisiae* 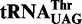 confers tRNA recognition by *Ag*AlaRS. Our work highlights the dynamic feature of natural genetic codes in mitochondria, and the relative simplicity by which tRNA identity may be switched.

## INTRODUCTION

When the genetic code was first deciphered in the 1960s, it was considered to be universal, with all organisms using the same standard code. Later it was shown that several codons have been recoded with different amino acids, thus creating non-standard genetic codes that are present in modern organisms (1). To date, 11 codon reassignment events have been reported in the nuclear genomes of bacteria, archaea and eukaryotes, and 16 have been found in mitochondria [reviewed in (1–4)]. Most recently, single-cell sequencing and biochemical analyses identified yet another, UGA tryptophan-to-glycine reassignment event in SR1 bacteria (5). These dogma-breaking discoveries suggest that the genetic code is evolvable in nature, and that it could be engineered in synthetic organisms. Enabled by genome engineering technologies, such as multiplex automated genome engineering (6), conjugative assembly genome engineering (7) and *de novo* genome synthesis (8), editing and rewriting the genetic code have emerged as an exciting topic in synthetic biology. Multiplex automated genome engineering, and conjugative assembly genome engineering have been used to change all 321 known UAG stop codons to the synonymous UAA stop codon. This will enable the abolition of UAG function, thereby permitting subsequent reassignment from ‘stop’ to any natural or non-natural amino acid (7).

Natural codon reassignment events may be explained by different evolutionary scenarios, depending on special circumstances. The codon capture mechanism (9) evokes that a specific set of codons and the corresponding tRNA completely disappear from a genome before a novel tRNA evolves to read such codons with a different specificity. In contrast, the ambiguous intermediate mechanism (10) posits that a codon does not need to disappear before reassignments and that it is ambiguously translated into distinct amino acids. More recently, reassignment scenarios have been discussed within an extended gain–loss framework of codons and tRNAs, pointing out that it is necessary to consider the details of each case carefully (11).

Codon reassignment is facilitated in mitochondrial DNAs (mtDNAs) because they encode only a small set of proteins, and tend to be A + T rich, which introduces a strong codon bias. For instance, UAA stop codons are highly preferred over UGA and UAG, making the latter available for reassignment from ‘stop’ to ‘sense’. The use of UGA (tryptophan) has evolved several times independently in the mitochondria of unrelated eukaryotic lineages, so has the introduction of UAG (Leu or Ala) [for a review see (12)]. Not only may stop codons be assigned to amino acids, but sense codons [e.g. UCA in *Scenedesmus obliquus* (13,14), and UUA, UUG in *Pycnococcus provasolii* (15)] may also convert to stop. It is interesting that UCA codons are absent in mitochondrial genes of close relatives of *S. obliquus* [i.e. *Chlamydomonas* and *Pedinomonas* (14)], in support of a codon capture mechanism in this case. Finally, sense codons may switch identity from one amino acid to another. The principle is similar to stop codon reassignment and facilitated when amino acids are encoded by alternative codon families, Leu (CUN or UUA/UUG in the standard code; N denotes A, U, G or C), Arg (CGN or AGA/AGG) and Ser (UCN or AGU/AGC). The less frequently used codon family may disappear, either completely or with only few remaining codons in non-critical sites of proteins, to allow switching of the aminoacyl-tRNA synthetase (aaRS) recognition site of a given tRNA to a different identity. Alternatively, extended phases of codon ambiguity may exist. For example, mutation of the tRNA^Ser^ anticodon to CAG and an m^1^G37 modification confer recognition of this tRNA by both seryl- and leucyl–tRNA synthetases, which is responsible for the ambiguous decoding of CUG by Ser and Leu in *Candida albicans* (16,17).

In this context, mitochondria are a special case. Their genes tend to be A + T rich with A or U preferred in third codon positions, and with genomes encoding small gene sets, which together simplifies liberation of codons for subsequent identity switch. However, an unmodified U in the wobble position of a tRNA’s anticodon is able to accommodate any of the four standard nucleotides in the codon’s third position (18,19), reducing the number of tRNAs that are required to recognize all codons to only 25 or even less [reviewed in (12,20)]. Consequently, switching identity of mitochondrial four-codon families by a codon capture mechanism requires liberation of the complete codon family before codon reassignment, which explains why so far only one such case has been identified. This example is the reassignment of CUN codons from Leu to Thr in the mitochondria of *Saccharomyces cerevisiae* (21,22), which belongs to a group of yeast species (Saccharomycetaceae) that lost all seven standard mitochondrial genes encoding subunits of the NADH dehydrogenase complex. Responsible for the reassignment event in the remaining eight protein-coding genes of *S. cerevisiae* is an unusual 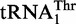 with a UAG anticodon (23), and an 8- rather than 7-nt-long anticodon loop, which has evolved from a tRNA^His^ ancestor (21). Here, we report a second instance in which a tRNA closely related to 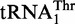 reassigns CUU and CUA codons in the mitochondrial genome of the yeast *A*. *gossypii* to Ala. We further provide preliminary evidence for a third event in another yeast species (*Nakaseomyces bacillisporus*), in which CGA is read as histidine rather than arginine.

## MATERIALS AND METHODS

### Cloning, mutagenesis and general methods

The *A*. *gossypii* AlaRS gene was cloned into pET28a expression vector (Novagen) with an N-terminal six-His tag. Expression of recombinant proteins was induced at 37°C for 4 hours with 0.5 mM isopropyl β-D-1-thiogalactopyranoside in *Escherichia coli* strain BL21-codon plus in Luria–Bertani media. His-tagged proteins were purified according to the standard procedures. Mitochondrial tRNA genes were cloned into pUC18 vector (GenScript), and mutations were introduced using QuikChange Site-Directed Mutagenesis Kit (Stratagene).

### *In vitro* assays with tRNAs

*In vitro* tRNA transcripts were obtained using the T7 RNA polymerase runoff procedure as described (24). Aminoacylation experiments were performed as described (25) in the presence of 100 mM Na-HEPES, pH 7.2, 30 mM KCl, 10 mM MgCl_2_, 2 mM ATP, 25 µM [^14^C] Ala or [^14^C] Thr, 5 µM tRNA transcripts and 30-3000 nM aaRSs.

### Identification of mitochondrial tRNAs in RNA-Seq data

*A. gossypii* cells (ATCC 10895) were grown to an optical density of ∼2.5 in a medium containing 1% yeast extract, 0.5% glucose and 3% glycerol, pH 5.5. Purification of a crude mitochondrial fraction followed previously published procedures (26), and RNA-Seq sequences (Illumina MiSeq; provided by the Genome Quebec Innovation Center) were generated from total, Trizol-extracted mitochondrial RNA, without size selection. Sequences in fastq format were quality-trimmed (phred 20, minimum sequence length 20 nt) and adapter-clipped using Seqtrimnext (http://rubygems.org/gems/seqtrimnext). tRNA sequences were analyzed by scanning the trimmed sequences with a 15-nt-long sequence window (using the basic Linux tools grep and wc).

### Identification of mitochondrial proteins by mass spectrometry

Purified mitochondria were solubilized in a buffer containing 20 mM Hepes/KOH, pH 7.4, 60 mM NH_4_Cl, 10 mM MgCl_2_, 0.5 mM ethylenediaminetetraacetic acid, 1 mM phenylmethylsulfonyl fluoride and digitonin (2 g/g of protein), followed by incubation on ice for 30 min and homogenization in a Potter homogenizer. After centrifugation at 18 000 *g* for 15 min, the supernatant was collected, and a small fraction (∼150 µg protein) was separated for 30 min (4–14% Blue Native Poly-Acrylamide Gel Electrophoresis (BN-PAGE); Hoefer apparatus with an 18 × 16 cm electrophoresis chamber; 140 V and 9 mA). The preparation of BN-PAGE gels, electrophoresis buffer and samples followed previously published procedures (27). The protein-containing zone was cut from the gel and submitted to liquid chromatography tandem mass spectrometry analysis (28,29), provided by a service platform at the Université de Montréal (Institute for Research in Immunology and Cancer). It includes destaining, reduction, alkylation, tryptic digestion and functional annotation by Mascot (30).

### Sequence alignments and identification of mitochondrial tRNAs

Derived mitochondrial protein sequences were aligned with Muscle version 3.6 (31). Mitochondrial tRNAs were identified with MFannot (http://megasun.bch.umontreal.ca/cgi-bin/mfannot/mfannotInterface.pl). It uses tool components of the Infernal package v1.1rc2 (32,33), notably cmbuild and cmcalibrate, to build a covariance search model from aligned tRNA training sets, followed by cmsearch to screen genomic sequences. The tRNA sequences shown in Figures 2 and 6 were aligned with cmsearch and the –A switch, using the standard tRNA model of MFannot. For visualization, editing and reformatting of sequence alignments, we used the Genetic Data Environment (GDE) sequence editor (34). A modified GDE version that functions with current 64-bit Linux versions, together with the appropriate libraries, is available on request.

#### Phylogeny of yeast species based on mtDNA-encoded protein sequences

Thirteen standard, mtDNA-encoded derived protein sequences (Cob, Cox1,2,3, Atp6,9 and Nad1,2,3,4,4L,5,6) were aligned in two steps. Briefly, sequences were pre-aligned with Muscle (31) and then refined with HMMalign (S. Eddy; http://hmmer.janelia.org). Sequence positions that were not aligned with a posterior probability value of 1.0 are discarded, and alignments were concatenated. The final dataset includes 40 species and has 3583 amino acid positions. The phylogenetic analysis was performed with PhyloBayes (35), the CAT/GTR model, six discrete categories, four independent chains, 14 000 cycles (corresponding to ∼770 000 generations) and the –dc parameter to remove constant sites. The first 10 000 cycles were discarded as burn-in.

#### Phylogeny of yeast mitochondrial tRNAs

The phylogenetic analysis with PhyloBayes (CAT, GTR, six categories; four independent chains, 11 000 cycles corresponding to ∼1 800 000 generations) contained all tRNA sequences from the species presented in Figure 5. Only the section of the tRNA phylogeny covering the related 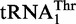 and tRNA^His^ clusters is shown in Figure 3.

## RESULTS

### Bioinformatic analyses suggest that CUU and CUA codons are reassigned from Leu to Ala in mitochondria of *A*. *gossypii*

**Figure 1.**
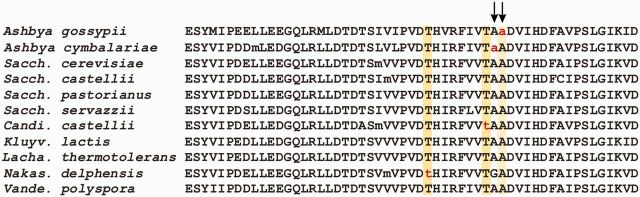
Alignment of derived Cox2 protein sequences and mass spectrometry at the protein level indicate that CUN decodes alanine in *A. gossypii*. The alignment corresponding to amino acids 141–195 of *A. gossypii* Cox2 is shown. Amino acids corresponding to CUN codons are in lower case, and translated into either Thr (most Saccharomycetaceae) or Ala (*A. gossypii*, confirmed by mass spectrometry of mitochondrial proteins. The sequence of the respective tryptic peptide is FIVTAaDVIHDFAVPSLGIK). Lower case m corresponds to AUA codons. This conservation pattern is valid for all mtDNA-encoded proteins of the shown species. Abbreviations: *Sacch*., *Saccharomyces*; *Candi.*, *Candida*; *Kluyv*., *Kluyveromyces*; *Lacha*., *Lachancea*; *Nakas.*, *Nakaseomyces*; and *Vande.*, *Vanderwaltozyma*.

Multiple sequence alignment of derived mitochondrial protein sequences reveals numerous positions where *Ashbya* does not conform to otherwise highly conserved, or even invariant, amino acids. For instance, in the given example of a cytochrome oxidase Cox2 sequence alignment (Figure 1), three columns containing CUN-encoded amino acids (amino acid shown in lower case) are highlighted. In the first two marked sequence columns, the pattern of conservation is consistent with translation of CUN to Thr (indicated by a lower-case t) for the given species, as experimentally confirmed in *S. cerevisiae* (21,22). In the third column, CUN corresponds to Ala in *A*. *gossypii* (shown as a lower-case a). This trend applies to all eight regular mtDNA-encoded proteins in *Ashbya* (49 CUA and 32 CUU codons; Supplementary Table S1), with most deviations in less well-conserved amino acid positions.

In the context of codon reassignment, the strict avoidance of CUG and CUC codons seems noteworthy. An analysis of overall mitochondrial codon usage reveals that *Ashbya* is as biased in other codon families, with 24 sense codons unused, most of which with a C or G in the third position (Supplementary Table S1). In other yeast species such as *S. cerevisiae*, overall codon bias is similar, although somewhat less extreme (Supplementary Table S1). A notable exception is in *Kluyveromyces lactis*, a close relative of *Ashbya*. This species avoids CUN codons altogether (Supplementary Table S1), and has no corresponding mtDNA-encoded tRNA with a UAG anticodon that would allow the recognition of this codon family.

### Mass spectrometry confirms the identity of CUU and CUA codons in *A. gossypii* mitochondrial proteins

To confirm the predicted CUA/CUU codon identity in *A. gossypii* as Ala, tryptic digests of proteins extracted from purified mitochondria were analyzed by mass spectrometry. To identify potential translation variants, results were analyzed based on three sets of inferred proteins in which CUA/CUU was translated as Leu, Thr or Ala. Among 475 identified proteins, two (Cox1 and Cox2) were mtDNA-encoded, with peptides covering gene regions with one CUA codon each. In both cases (experiment repeated three times), CUA was translated as Ala but not Thr or Leu (for mass spectrometry data of identified Cox2 peptides, see Supplementary Figure S1). Protein regions translated with CUU codons were not identified.

### *In silico* identification of the *A. gossypii* tRNA decoding CUU and CUA codons

**Figure 2.**
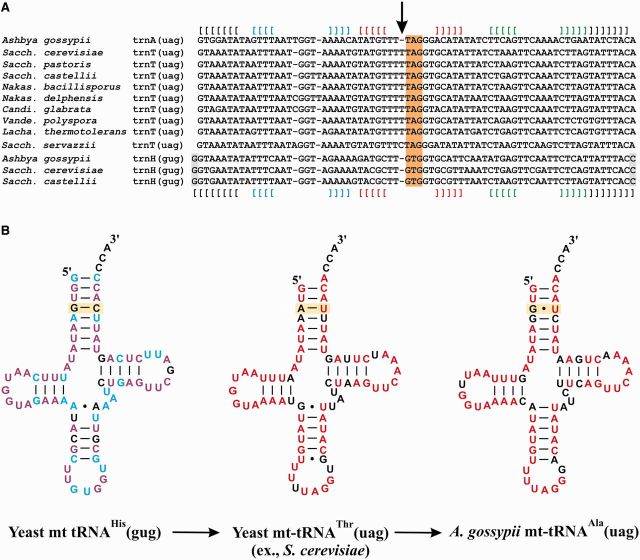
Primary and secondary structures of yeast mitochondrial 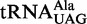, 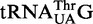 and 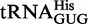. (**A**) Selected yeast mitochondrial tRNAs with UAG or GUG anticodons (anticodon marked orange) are aligned (upper and lower blocks of sequences, respectively). Square brackets indicate the four standard helical regions in tRNAs. The arrow points to a nucleotide insertion that leads to a characteristic 8-nt anticodon loop in 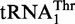 (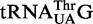). In *A. gossypii*, the anticodon loop has 7 nucleotides, and this tRNA reads CUN codons as Ala but not Thr. As 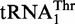 is most likely derived from tRNA^His^ by duplication, tRNA^His^ is included in the alignment for comparison [see also (21)]. Note the characteristic G residue at position −1 (constituting the 5′-terminus of tRNA^His^) that pairs with the C at the 3′-discriminator position (both positions marked in gray). (**B**) Secondary structures of tRNAs that illustrate a possible tRNA reassignment scenario, *S. cerevisiae* 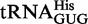 (left), *A. gossypii* 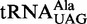 (right) and *S. cerevisiae* 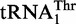 (middle). Nucleotide identity is coded as follows: red, *A. gossypii* 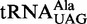 *versus S. cerevisiae* 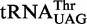; magenta, identity across all three tRNAs; and blue, additional identity between *A. gossypii* and *S. cerevisiae* 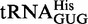. Following a codon capture mechanism, 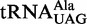 and 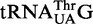 would have evolved from a 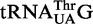 because the latter is present in the sister lineage *Lachancea* species (for a species phylogeny, see Figure 5), and absent in *K. lactis*. For abbreviations, see Figure 1.

**Figure 3.**
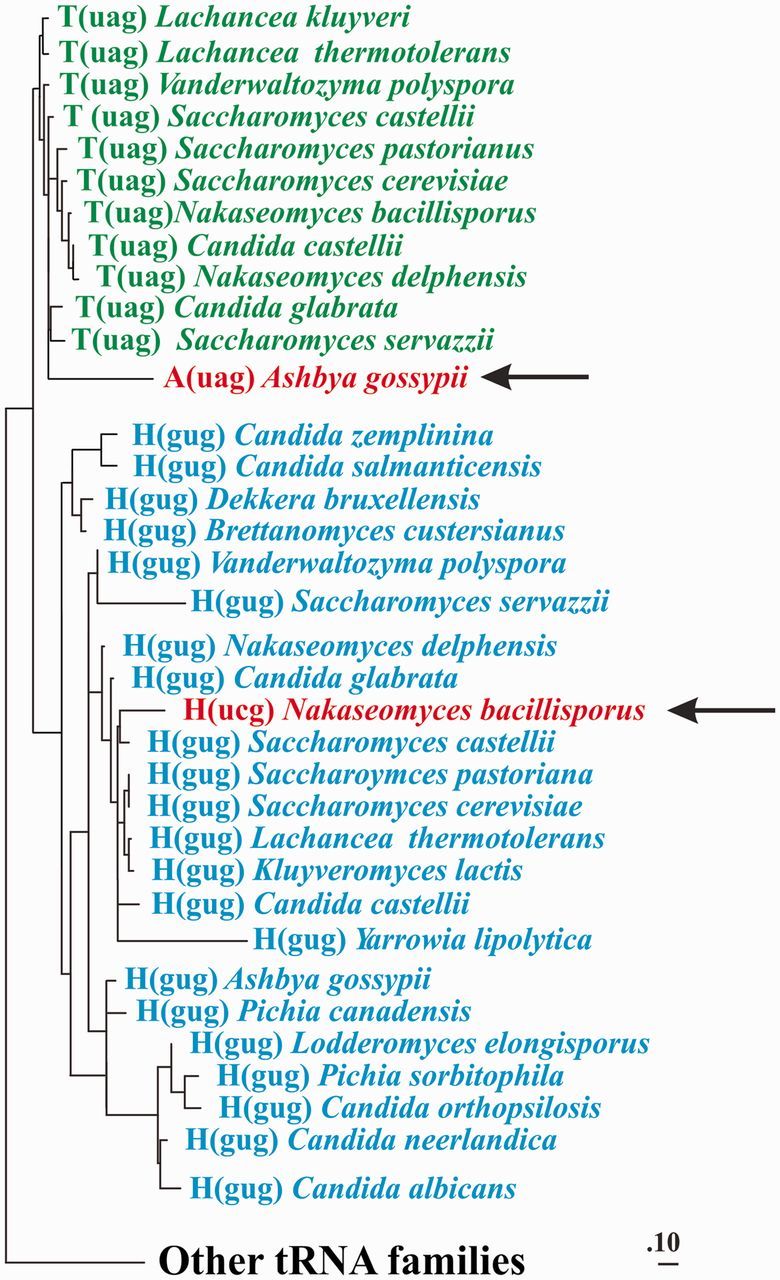
Phylogeny of yeast mitochondrial tRNAs. The phylogenetic analysis contains representatives from a broad selection of yeast species. Only the section of the tree in which relevant 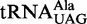, 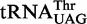 and 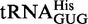 cluster together is shown. The posterior probability support for the two tRNA groups is indicated. Green and blue indicate 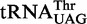, and 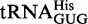 families, respectively. Red indicates reassigned tRNAs. Note that phylogenetic analysis with tRNA sequences depends on only a few informative nucleotide positions, which does not allow resolving the branching order within these groups.

Identification of yeast mitochondrial tRNAs is highly sensitive and without false positives when using covariance search models (32), which are used by our annotation tool MFannot (12). All known tRNA structures with a UAG anticodon (tRNA_UAG_) were identified throughout yeast species (e.g. Figure 2A), with the notable exception of *K. lactis*, as mentioned earlier in the text. In distinction to the 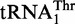 in *S. cerevisiae* and most other Saccharomycetaceae, the *A. gossypii* tRNA_UAG_ has a standard 7-nt anticodon loop (Figure 2A). A comparison of tRNA_UAG_ across yeast species further reveals >70% sequence identity (e.g. Figure 2A and B), pointing to a recent common ancestry.A phylogenetic analysis of mtDNA-encoded tRNAs confirms this view, clustering 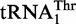 of Saccharomycetaceae with *A. gossypii* tRNA_UAG_ (Figure 3). This analysis further confirms that tRNA_UAG_ from yeast mitochondria was originally derived from a duplication of a histidine tRNA, as previously proposed (21). The tRNA phylogeny further reveals an unexpected clustering of *N. bacillisporus* mitochondrial tRNA(UCG) within 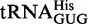 (Figure 3). A more detailed comparison with histidine tRNAs (Figure 6) reveals a shared recognition signal for histidyl-tRNA synthetase (HisRS) (17), a G residue at position −1 (the 5′ terminus of tRNA histidine) that pairs with a C at the 3′ discriminator position. This suggests that tRNA (UCG) might recognize the CGN codon family not as arginine (see Discussion).

To demonstrate that *A. gossypii* tRNA_UAG_ is expressed, properly processed and matured with a 3′-CCA terminus, RNA-Seq data from total RNA were produced and analyzed. This tRNA is expressed at about the same level as other mitochondrial tRNA species. The 5′- and 3′-processing intermediates and mature tRNA (with CCA addition at the 3′) were confirmed. The sequence of tRNA_UAG_ is identical to that of the genomic DNA (including the anticodon), suggesting that the tRNA is not edited post-transcriptionally (36).

### CUU/CUA-decoding tRNA in *A. gossypii* mitochondria is recognized by the mitochondrial alanyl-tRNA synthetase

**Figure 4.**
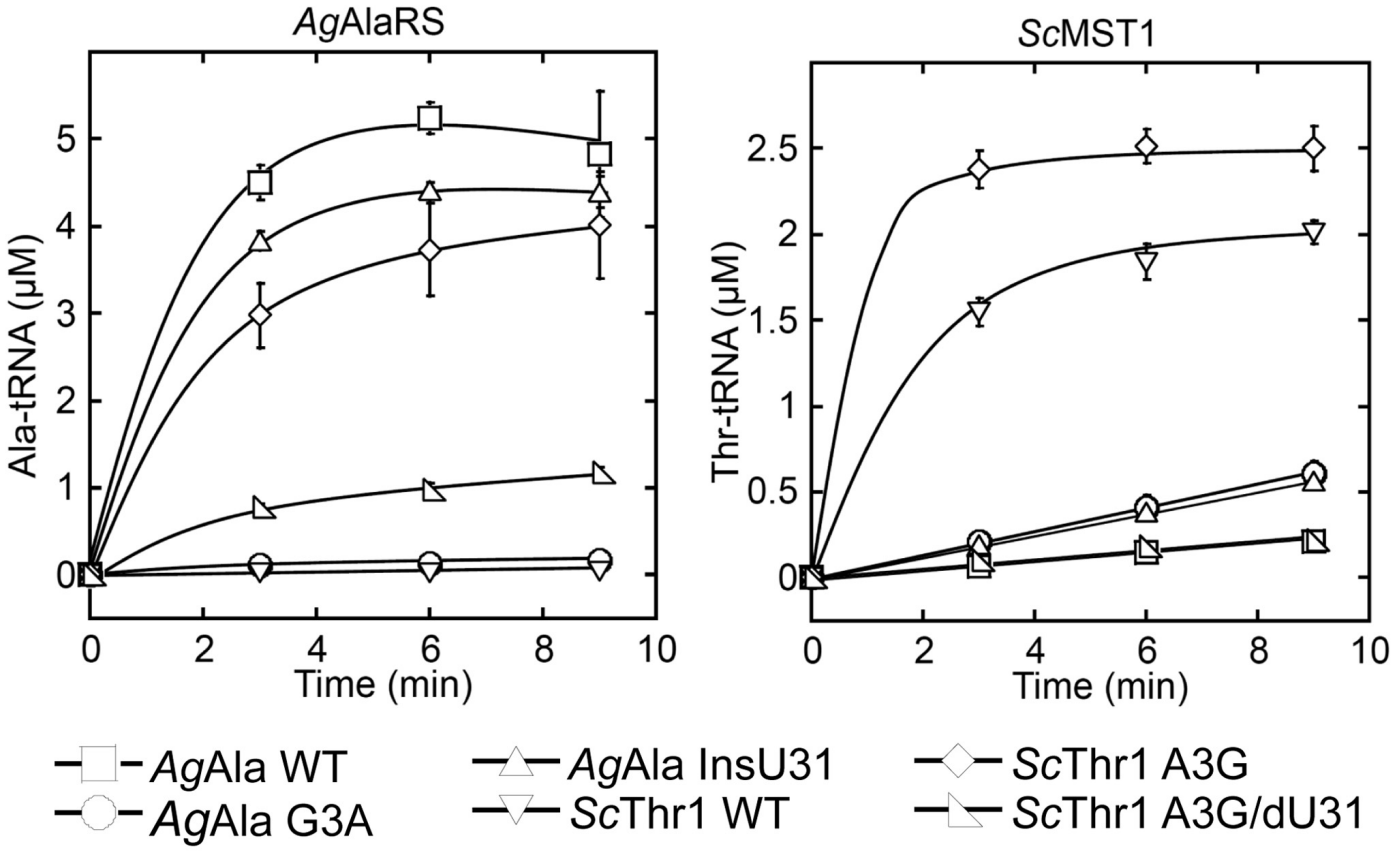
Aminoacylation of mitochondrial tRNA^Ala^ and tRNA^Thr^ variants by *Ag*AlaRS and *Sc*MST1. The reaction was performed at 37°C in the presence of 3 µM *Ag*AlaRS or *Sc*MST1, 5 µM tRNA transcript and 25 µM [^14^C]Ala or [^14^C]Thr.

The tRNA with a predicted UAG anticodon in *A. gossypii* mitochondria is closely related to *S. cerevisiae* mitochondrial 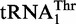 (Figures 2B and 3). Therefore, we tested whether the *A. gossypii* mitochondrial tRNA_UAG_ is a substrate for *Sc*MST1, which is the enzyme responsible for attaching Thr onto *S. cerevisiae* mitochondrial 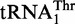. *In vitro* aminoacylation experiments show that the wild-type (WT) *A. gossypii* mitochondrial tRNA_UAG_ is not recognized by *Sc*MST1 (Figure 4). Previously we showed that the enlarged 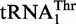 anticodon loop is an important identity element for *Sc*MST1 (21,37). In line with that, a U insertion (InsU31) in the anticodon loop of *A. gossypii* mitochondrial tRNA_UAG_ converts it to a moderate substrate for *Sc*MST1 in an aminoacylation experiment with Thr (Figure 4). To further validate the identity of this tRNA_UAG_ species, we purified the recombinant *A. gossypii* mitochondrial AlaRS (*Ag*AlaRS) and tested Ala acylation. The WT tRNA_UAG_ from *A. gossypii* mitochondria turned out to be a good substrate for *Ag*AlaRS, and was therefore named 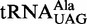.

### The G3:U70 pair of 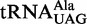 is critical for reassignment of CUU and CUA codons as Ala

The major identity element for AlaRS enzymes is the G3:U70 base pair (38,39). This pair is also present in *A. gossypii* mitochondrial 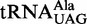. A G3A change in 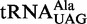 abolishes its recognition by *Ag*AlaRS (Figure 4), suggesting that *Ag*AlaRS recognizes its tRNA substrate in a similar manner as the characterized AlaRSs. As expected, the WT *S. cerevisiae* mitochondrial 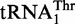, which contains an A3:U70 pair, is not a substrate for *Ag*AlaRS. However, an A3G mutant of *S. cerevisiae* mitochondrial 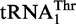 gains 12% alanylation efficiency compared with the WT *A. gossypii* mitochondrial 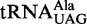 (Figure 4 and Table 1). Further, deletion of U31 in the A3G/dU31 mutant prevented the tRNA from recognition by MST1. These results suggest that *A. gossypii* mitochondrial 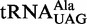 has evolved to an orthogonal Ala tRNA not recognized by ThrRS, either via duplication of 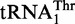 or its evolutionary precursor tRNA^His^ (21). The emergence of a G3:U70 pair would be the defining evolutionary step during this reassignment process.

**Table 1.** Aminoacylation efficiency of tRNA variants by *A. gossypii* AlaRS

tRNA	*k*_cat_ (min^−1^)	*K*_m_ (µM)	*k*_cat_/*K*_m_ (µM^−1^ min^−1^)	Relative *k*_cat_/*K*_m_
tRNA^Ala^ WT	2.1 ± 0.4	0.53 ± 0.35	5.3 ± 3.0	100
tRNA^Ala^ InsU31	2.1 ± 0.2	1.0 ± 0.4	2.2 ± 0.8	42
tRNA^Thr^ A3G	1.0 ± 0.04	1.6 ± 0.08	0.65 ± 0.03	12

## DISCUSSION

### Reassignment of CUN codons in yeast mitochondria

**Figure 5.**
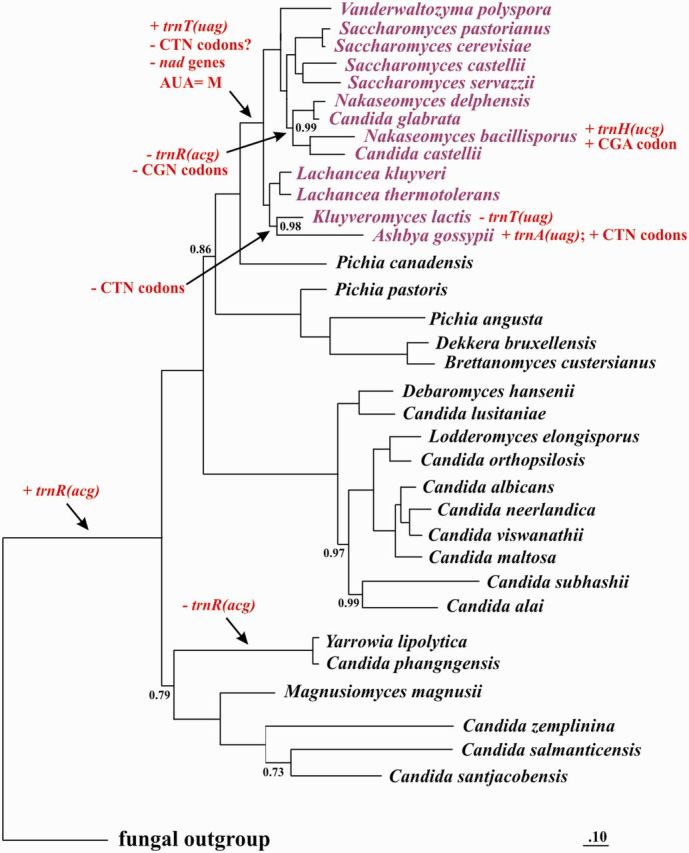
Evolution of yeast species and codon reassignment events. The phylogenetic analysis with PhyloBayes and the CAT/GTR model is based on 13 mtDNA-encoded proteins. All divergence points are supported by posterior probability values of 1.0, except where indicated; Saccharomycetaceae are in magenta. The fungal outgroup consisted of *Rhizopus oryzae*, *Aspergillus niger*, *Podospora anserina*, *Fusarium oxysporum*, *Cantharellus cibarius* and *Ustilago maydis.* All mitochondrial sequences were downloaded from the ‘Organelle Genome Resources’ Web site of NCBI. The red arrow points to the concomitant loss of all seven *nad* genes (subunits of NADH dehydrogenase complex) and the start of major mitochondrial codon reassignments, including AUA methionine, CUN threonine, CUN alanine and (most unusual) CGN histidine.

The mitochondrial genetic code has been rapidly evolving across eukaryotes, including a relatively recent CUN codon reassignment close to the divergence of the Saccharomycetaceae family (Figure 5). This group of yeast species has reduced mitochondrial gene numbers substantially (no *nad* genes, only 7–8 protein-coding genes are left in Saccharomycetaceae), which implies that codon families can be even more easily eliminated than in other mitochondrial systems. For example, *K*. *lactis* (a close relative of *Ashbya*) does not use CUN codons (Supplementary Table S1) and has no mtDNA-encoded tRNA with a UAG anticodon that would allow the recognition of this codon family. Based on the combined evidence, we favor the interpretation that CUN codon reassignment followed a codon capture mechanism, where CUN codons and the corresponding tRNA first vanished in the mitochondrial genome (completely, or more likely, reduced to a few codons that have little impact on protein structure and function), followed by the emergence of a tRNA with UAG anticodon that decodes CUN as alanine and the reappearance of more CUN codons (9,21). A short period of coding ambiguity cannot be ruled out if this new tRNA arose by duplication. However, a single mutation at the aaRS recognition site would suffice for an identity switch from threonine to alanine, without codon ambiguity. We favor this latter interpretation over other alternatives that may rather apply to more complex genetic systems [e.g. the nuclear genome of *C*. *albicans* (3)] than to yeast mitochondria with much fewer protein-coding genes.

Our previous work reveals that in *Saccharomyces*, 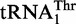, which is responsible for CUN codon reassignment from Leu to Thr, was derived from a mitochondrial tRNA^His^ (21). Loss of CUN codons (accompanied by loss of *nad* genes) and evolution of 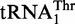 from tRNA^His^ *via* gene duplication presumably occurred in a common ancestor of *Saccharomycetaceae* (Figure 5). Gene duplications are common in mtDNA. For example, among extant yeast species, the *C*. *albicans* strain SC5314 mitochondrial genome encodes two copies of tRNA^His^ within duplicated genome segments—the basis for neo-functionalization of one of the gene copies (40).

In the current work, we show that *A*. *gossypii* mitochondria use CUA and CUU to decode Ala instead of Leu or Thr. 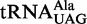 clearly clusters with yeast mitochondrial 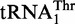, from which it most likely also derived *via* gene duplication. This reassignment implies a reduction of the unusual 8-nt anticodon loop (the recognition site of threonyl–tRNA synthetase) to only seven nucleotides, and introduction of a G3:U70 base pair, which allows efficient recognition of CUN codons by *A. gossypii* mitochondrial alanyl-tRNA synthetase (Figure 2).

### Evolution of mitochondrial 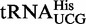 from 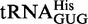 in *N*. *bacillisporus*

**Figure 6.**
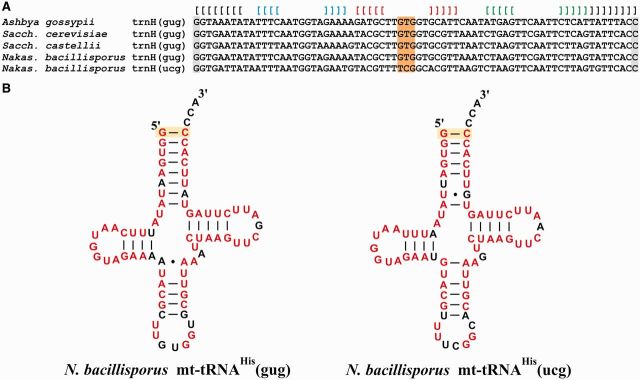
Primary and secondary structures of *N. bacillisporus* mitochondrial 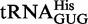 and putative 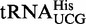. (**A**) Selected yeast mitochondrial tRNAs^His^ with GUG anticodons (anticodon marked orange) are aligned in comparison with an unusual, predicted *N. bacillisporus* mitochondria tRNA^His^ with a UCG anticodon (that according to the standard genetic code would recognize CGN arginine). Square brackets indicate the four typical helical regions in tRNAs. Note the characteristic G residue at position −1 (constituting the 5′ terminus of tRNA histidine) that pairs with the C at the 3′ discriminator position (both positions marked in gray). (**B**) Secondary structures of the two mitochondrial *N. bacillisporus* histidine tRNAs. Nucleotide identity is coded in red, and the recognition sequence for HisRS (G–C base pair at position −1) is marked yellow.

The tRNA phylogeny further reveals a divergence of a *N. bacillisporus* mitochondrial tRNA(UCG) from within the 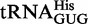 cluster (Figure 3). According to the standard translation code, a tRNA with this anticodon is expected to recognize the CGN arginine codon family. Yet, based on the following considerations, we predict instead that it most likely translates CGA as His: (i) 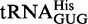 and 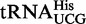 in this species are 86% identical at the primary sequence level (Figure 6), (ii) both tRNAs have a gene-encoded G at position −1, and a C in the discriminator position, forming a base pair that is the known recognition signal for HisRS (21), (iii) CGN codons do not exist in neighbor species of *N. bacillisporus* (*Nakaseomyces delphensis*, *Candida glabrata* and *Candida castellii*; Figure 5), preparing the way for CGN codon capture and (iv) *N. bacillisporus* has a single CGA codon in the *cox1* gene, at an overall poorly conserved amino acid position. Remarkably, its closest neighbor *C. castellii* has a histidine in this sequence position that is overall not well conserved. An identity switch of the unique CGA codon from arginine to histidine would therefore have little, if any, functional bearing, but as *N. bacillisporus* HisRS has to recognize the GUG anticodon of 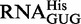 (21), a specificity change of HisRS is required to also recognize CGA (or alternatively, by a duplicated and modified HisRS). Whether this implies a transition period with ambiguous codon recognition depends on the facility with which mutations become established. Further experimental evidence is needed to validate our prediction that CGA is reassigned from Arg to His in *N. bacillisporus* mitochondria.

### Natural evolution of orthogonal tRNAs

According to our interpretation, CUN codon identity in yeast mitochondria has evolved in the order Leu to Thr to Ala, with corresponding specific tRNAs for Thr and Ala deriving from duplicates of 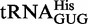 and 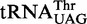, respectively (Figure 2B). The suggested complete reassignment of these codons requires that tRNAs are orthogonal to the corresponding aaRS (recognized by a single cognate aaRS). In other words, recognition of a tRNA by more than one aaRSs would result in ambiguous decoding, as observed in the nuclear genome of several *Candida* species, where CUG is read by both Leu and Ser (16,41).

Experimental data and the presence of known tRNA identity signatures are consistent with our hypothesis. Mass spectrometry identified Ala (not Leu, His or Thr) in highly conserved positions of *A. gossypii* mtDNA-encoded proteins, corresponding to CUA at the codon level and suggesting that 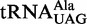 is orthogonal to AlaRS. We further show experimentally that 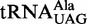 is not recognized by *S. cerevisiae* MST1, in line with the previous observation that this enzyme recognizes the extended 8-nt-long anticodon loop of 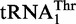 (37). Most decisively, *A. gossypii* 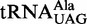 has a G3:U70 base pair, which is a known identity signature for AlaRS (38,42). Recognition of this tRNA by mitochondrial HisRS can be excluded, as it is known to recognize G_−__1_ (a nucleotide at position −1 according to standard nomenclature that is not present in other tRNAs), the anticodon GUG and the discriminator base C73 (21). *A. gossypii* 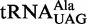 has all of these identity elements altered, and is therefore unlikely to be a substrate for HisRS. Finally, this tRNA has the same anticodon as mitochondrial tRNA^Leu^ outside Saccharomycetaceae. However, leucyl–tRNA synthetases typically recognize A73 (not present in *A. gossypii* 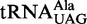) and not the anticodon (37). Thus, we conclude that CUA and CUU are decoded solely by Ala in *A. gossypii* mitochondria.

Studying sense codon recoding is not only important for understanding the evolution of life, but also provides valuable insights into manipulation of the genetic code to create synthetic organisms. One roadblock of sense codon recoding is the orthogonality of the synthetic tRNA (Krishnakumar *et al.*, submitted for publication), which requires that the synthetic tRNA is not recognized by any endogenous aaRS. Identifying naturally evolved orthogonal tRNAs would thus offer insights into the principles that have to be followed, and the translational components that need to be adapted, for recoding the translation machinery of synthetic organisms.

## SUPPLEMENTARY DATA

Supplementary Data are available at NAR Online, including [43].

## FUNDING

The National Institute of General Medical Sciences [GM022854 to D.S.], FQRNT and the National Research Council [NSERC to B.F.L.], the Canadian Research Chair Program (to B.F.L.) and the Department of Energy [DE-FG02-02ER63445 to G.C.]. Funding for open access charge: NIH [GM022854].

*Conflict of interest statement*. None declared.

## Supplementary Material

Supplementary Data
