# Supplementary material for: Natural reassignment of CUU and CUA sense codons to alanine in Ashbya mitochondria
Source: Nucleic Acids Res. 2013 Sep 17;42(1):499–508. doi: 10.1093/nar/gkt842 (PMC3874161; doi:10.1093/nar/gkt842)
Supplement: Supplementary Data [file supp_42_1_499__index.html]

Natural reassignment of CUU and CUA sense codons to alanine in Ashbya mitochondria — Natural reassignment of CUU and CUA sense codons to alanine in Ashbya mitochondria — Supplementary Data 

# Natural reassignment of CUU and CUA sense codons to alanine in *Ashbya* mitochondria

## Supplementary Data

files

**Files in this Data Supplement:**

- Supplementary Data - docx file
