# Supplementary material for: Natural reassignment of CUU and CUA sense codons to alanine in Ashbya mitochondria
Source: Nucleic Acids Res. 2013 Sep 17;42(1):499–508. doi: 10.1093/nar/gkt842 (PMC3874161; doi:10.1093/nar/gkt842)
Supplement: Supplementary Data [file supp_gkt842_nar-01955-r-2013-File002.docx]

**Supplementary Information**

**Table S1: Mitochondrial codon usage of *A. gossypii*, *S. cerevisiae*, *L. thermotolerans*, and *K. lactis*.** Codon usage tables were inferred based on the standard genes *cob*, *cox1,2,3*, *atp6,8,9* and *rps3*. The inferred CUN codon identity is indicated.

***Ashbya gossypii* (CUA, CUU, alanine)**

**========================================**

**F UUU 106 S UCU 40 Y UAU 121 C UGU 15**

**F UUC 43 S UCC 0 Y UAC 2 C UGC 0**

**L UUA 288 S UCA 71 * UAA 7 W UGA 37**

**L UUG 1 S UCG 0 * UAG 1 W UGG 0**

**========================================**

**A CUU 31 P CCU 36 H CAU 52 R CGU 0**

**A CUC 0 P CCC 0 H CAC 0 R CGC 0**

**A CUA 49 P CCA 43 Q CAA 46 R CGA 0**

**A CUG 0 P CCG 0 Q CAG 2 R CGG 0**

**========================================**

**I AUU 215 T ACU 65 N AAU 167 S AGU 31**

**I AUC 7 T ACC 0 N AAC 2 S AGC 0**

**M AUA 95 T ACA 33 K AAA 75 R AGA 40**

**M AUG 33 T ACG 0 K AAG 6 R AGG 0**

**========================================**

**V GUU 44 A GCU 50 D GAU 52 G GGU 121**

**V GUC 0 A GCC 0 D GAC 0 G GGC 0**

**V GUA 68 A GCA 17 E GAA 42 G GGA 9**

**V GUG 0 A GCG 0 E GAG 4 G GGG 0**

***Saccharomyces cerevisiae* (CUA, CUU, threonine)**

**========================================**

**F UUU 81 S UCU 39 Y UAU 106 C UGU 13**

**F UUC 67 S UCC 0 Y UAC 11 C UGC 1**

**L UUA 285 S UCA 78 * UAA 8 W UGA 38**

**L UUG 2 S UCG 0 * UAG 0 W UGG 1**

**========================================**

**T CUU 6 P CCU 46 H CAU 53 R CGU 1**

**T CUC 0 P CCC 3 H CAC 5 R CGC 0**

**T CUA 19 P CCA 36 Q CAA 36 R CGA 0**

**T CUG 0 P CCG 1 Q CAG 5 R CGG 1**

**========================================**

**I AUU 193 T ACU 40 N AAU 196 S AGU 25**

**I AUC 29 T ACC 1 N AAC 12 S AGC 0**

**I AUA 38 T ACA 53 K AAA 70 R AGA 42**

**M AUG 72 T ACG 0 K AAG 3 R AGG 0**

**========================================**

**V GUU 50 A GCU 66 D GAU 53 G GGU 99**

**V GUC 7 A GCC 7 D GAC 2 G GGC 1**

**V GUA 83 A GCA 54 E GAA 41 G GGA 31**

**V GUG 5 A GCG 4 E GAG 3 G GGG 6**

***Kluyveromyces lactis* (CUN unused)**

**========================================**

**F UUU 71 S UCU 35 Y UAU 100 C UGU 14**

**F UUC 90 S UCC 1 Y UAC 13 C UGC 1**

**L UUA 289 S UCA 98 * UAA 8 W UGA 40**

**L UUG 2 S UCG 1 * UAG 0 W UGG 0**

**========================================**

**- CUU 0 P CCU 49 H CAU 54 R CGU 0**

**- CUC 0 P CCC 4 H CAC 2 R CGC 0**

**- CUA 0 P CCA 35 Q CAA 48 R CGA 0**

**- CUG 0 P CCG 1 Q CAG 0 R CGG 0**

**========================================**

**I AUU 213 U ACU 41 N AAU 154 S AGU 27**

**I AUC 16 U ACC 0 N AAC 13 S AGC 2**

**I AUA 3 U ACA 66 K AAA 75 R AGA 46**

**M AUG 64 U ACG 0 K AAG 1 R AGG 1**

**========================================**

**V GUU 50 A GCU 73 D GAU 49 G GGU 86**

**V GUC 2 A GCC 4 D GAC 2 G GGC 1**

**V GUA 102 A GCA 50 E GAA 48 G GGA 54**

**V GUG 1 A GCG 3 E GAG 2 G GGG 2**

***Lachancea thermotolerans* (CUA, CUU, threonine)**

**========================================**

**F UUU 85 S UCU 41 Y UAU 93 C UGU 16**

**F UUC 72 S UCC 0 Y UAC 27 C UGC 0**

**L UUA 303 S UCA 82 * UAA 6 W UGA 41**

**L UUG 1 S UCG 0 * UAG 2 W UGG 0**

**========================================**

**T CUU 5 P CCU 56 H CAU 49 R CGU 2**

**T CUC 0 P CCC 1 H CAC 5 R CGC 0**

**T CUA 11 P CCA 27 Q CAA 47 R CGA 0**

**T CUG 0 P CCG 0 Q CAG 0 R CGG 0**

**========================================**

**I AUU 204 U ACU 39 N AAU 145 S AGU 30**

**I AUC 18 U ACC 0 N AAC 16 S AGC 3**

**M AUA 4 U ACA 63 K AAA 78 R AGA 44**

**M AUG 56 U ACG 0 K AAG 1 R AGG 0**

**========================================**

**V GUU 51 A GCU 88 D GAU 51 G GGU 82**

**V GUC 1 A GCC 5 D GAC 2 G GGC 0**

**V GUA 103 A GCA 50 E GAA 49 G GGA 53**

**V GUG 1 A GCG 0 E GAG 1 G GGG 2**


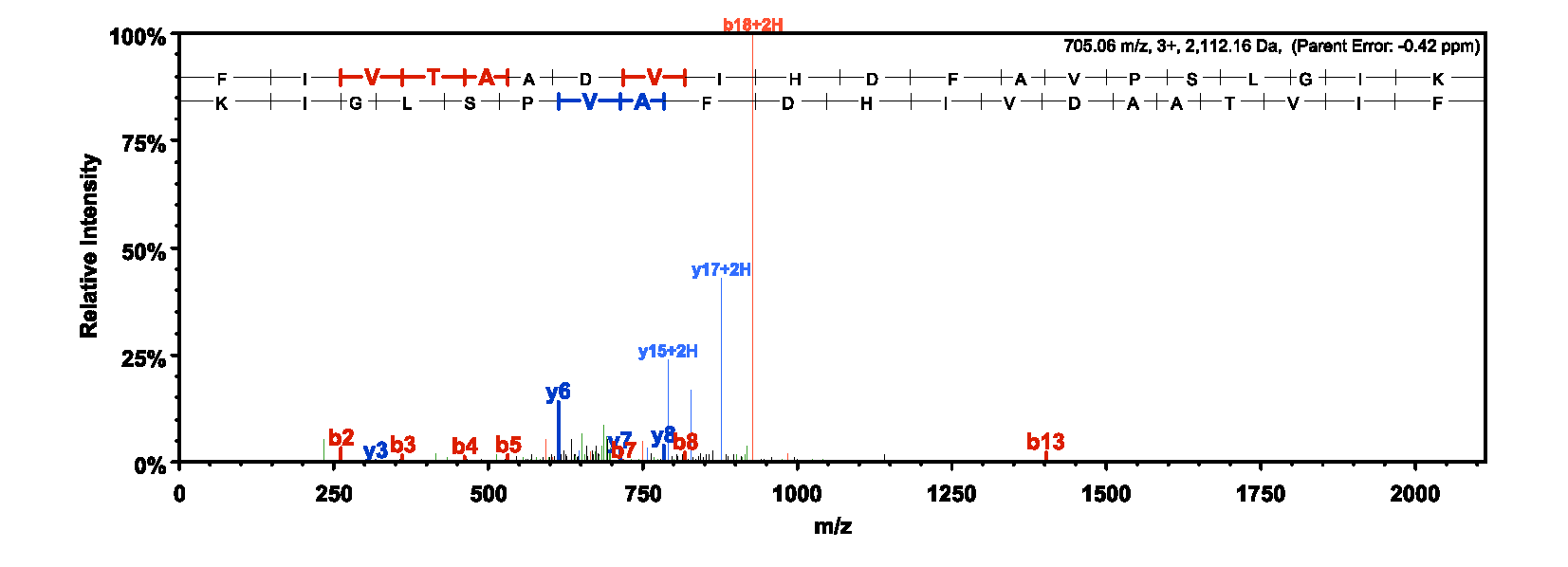


**Figure S1: MS/MS spectrum of *Ashbya gossypii* Cox2p tryptic peptides.** MS/MS spectrum assigned to a Cox2p peptide (FIVTAADVIHDFAVPSLGIK) containing a CUA codon, which is translated as alanine (position 6, underlined). The MS/MS spectra were manually reviewed using Scaffold ([1](#_ENREF_1)).

1. Searle, B.C. (2010) Scaffold: a bioinformatic tool for validating MS/MS-based proteomic studies. *Proteomics*, **10**, 1265-1269.
